# GLP-1 receptor agonist use in patients with psoriasis or hidradenitis suppurativa

**DOI:** 10.1016/j.jdin.2025.12.008

**Published:** 2025-12-26

**Authors:** Rachel Granovsky, Allison Bai, Ilona Ponyatyshyn, Zhuoqing Suzy Gellatly, Jatin Narang, Gabriela Cobos

**Affiliations:** a Tufts University School of Medicine, Boston, Massachusetts; b University of Illinois College of Medicine, Chicago, Illinois; c Department of Dermatology, Tufts Medical Center, Boston, Massachusetts

**Keywords:** glucagon-like peptide-1 receptor agonist, hidradenitis suppurativa, medical dermatology, metabolic dysfunction, obesity, psoriasis

*To the Editor:* Glucagon-like peptide-1 receptor agonists (GLP-1RAs) have a compelling use in dermatology given the strong association between metabolic dysfunction and conditions like psoriasis and hidradenitis suppurativa (HS).^1^ Case reports have demonstrated clinical improvements in HS and psoriasis severity following GLP-1RA treatment, including reductions in BMI, Psoriasis Area and Severity Index score, and Dermatology Life Quality Index.^2^ This study aimed to quantify GLP-1RA eligibility, counseling, and prescribing among adults with psoriasis or HS, and to assess whether gaps in care varied by demographic or health care access factors.

We conducted a retrospective chart review of adults who visited Tufts Medical Center Dermatology between January 1, 2020 and December 31, 2024, with a diagnosis of psoriasis or HS confirmed using ICD-10 codes and dermatology note review. GLP-1RA eligibility was defined per clinical guidelines^3^: (a) BMI >30 or (b) BMI >27 and a comorbidity including Type 2 Diabetes Mellitus, hyperlipidemia, obstructive sleep apnea, metabolic associated fatty liver, chronic kidney disease, or atherosclerotic heart disease.^3^ Data were collected via EHR review using ICD-10 codes to identify relevant diagnoses and a free text search for “GLP-1” or common brand names (“Ozempic,” “Wegovy,” “tirzepatide”) to identify documentation of GLP-1 counseling. Disease severity (Hurley Stage or Physician's Global Assessment (PGA)) was extracted from dermatology notes closest to BMI measurement.

Patient characteristics are summarized in Table I. Among 1490 patients (1144 psoriasis; 346 HS), patients HS patients were more often female (74%, *P* < .00001), younger (mean age difference = 15 years, *P* < .00001), non-White (40%, *P* < .00001), and on Medicaid (21.5%, *P* < .00001). Psoriasis patients were more often Asian (19.1%, *P* < .00001), on Medicare (25.6%, *P* < .00001), and non-English speaking (11.7%, *P* < .00001). GLP-1RA eligibility, counseling, and prescribing patterns are presented in Table II. Overall, 890 (59.7%) patients qualified for GLP-1RA therapy (54.9% psoriasis; 72.3% HS). Most 619 (70%) qualified based on BMI alone, while 268 (30%) qualified due to comorbidities. Only 324 (36.4%) of 890 eligible patients received counseling and 194 (22.1%) were prescribed a GLP-1RA. Most counseling and prescriptions were initiated by PCPs 209 (64.5%) or endocrinology 71 (21.9%). Only 2 patients (0.6%) were prescribed by dermatology. Notably, 102 (18%) of 566 eligible but uncounseled patients lacked a PCP; among these, 24 (23.5%) saw only dermatology and 1 other specialist.

**Table I:** Demographic characteristics of patients with psoriasis or hidradenitis suppurativa seeking care at Tufts Medical Center 2020 to 2024

	Psoriasis (*n* = 1144)	HS (*n* = 346)	Total (*N* = 1490)	*P*-value∗
Sex, *n* (%)				<.00001
Male	534 (46.7%)	90 (26.0%)	624 (41.9%)	
Female	610 (53.3%)	256 (74.0%)	866 (58.1%)	
Age, median (range)	52 (18–97)	37 (18–85)	50 (18–97)	<.00001
Race/Ethnicity, *n* (%):				<.00001
White	748 (65.4%)	146 (42.2%)	894 (60.0%)	
Black	66 (5.8%)	98 (28.3%)	164 (11.0%)	
Asian	219 (19.1%)	27 (7.8%)	246 (16.5%)	<.00001
Hispanic	78 (6.8%)	63 (18.2%)	141 (9.5%)	
Other/Unknown	33 (2.9%)	12 (3.5%)	45 (3.0%)	
Insurance, *n* (%):				<.00001
Commercial	658 (57.5%)	189 (54.6%)	847 (56.8%)	
Medicare	293 (25.6%)	30 (8.7%)	323 (21.7%)	<.00001
Medicaid	193 (16.9%)	127 (36.7%)	320 (21.5%)	
Primary language, *n* (%):				<.00001
English	1010 (88.3%)	336 (97.1%)	1346 (90.3%)	
Chinese†	76 (6.6%)	0	76 (5.1%)	
Spanish	17 (1.5%)	8 (2.3%)	25 (1.7%)	
Other‡	41 (3.6%)	2 (0.6%)	43 (2.9%)	

∗ *T*-tests used for numerical data and Chi squared for categorical.

† Chinese includes Cantonese, Mandarin, and Toishanese.

‡ Other includes Portuguese, Vietnamese, Russian, Haitian, Cape Verdean, and Arabic.

**Table II:** GLP-1 receptor agonist eligibility, counseling, and prescribing patterns in patients with psoriasis or hidradenitis suppurativa

	PS	HS	Total
Qualify for GLP	629 (54.9%)	250 (72.3%)	890 (59.7%)
Qualify based on BMI	401 (63.8%)	210 (84.0%)	619 (69.6%)
Qualify based 1 comorbidity	73 (11.6%)	25 (10.0%)	98 (11.0%)
Qualify based on 2 comorbidities	83 (13.2%)	5 (2.0%)	88 (9.89%)
Qualify based on 3 comorbidities	72 (11.4%)	10 (4.0%)	82 (9.21%)
GLP-1RA prescribing data			
Currently on GLP	131 (20.8% of pts who qualify)	62 (24.5% of pts who qualify)	194 (22.1% of pts who qualify)
Previously on GLP	31 (4.92%)	10 (4%)	41 (4.66%)
What drug	Tirzepatide: 65 (43.3%)	Tirzepatide: 27 (41.5%)	Tirzepatide: 92 (42.7%)
	Semaglutide: 63 (42%)	Semaglutide: 26 (41.9%)	Semaglutide: 89 (41.4%)
	Dulaglutide: 22 (14.66%)	Dulaglutide: 9 (13.8%)	Dulaglutide: 31 (14.4%)
	Liraglutide: 0	Liraglutide: 3 (4.62%)	Liraglutide: 3 (1.4%)
GLP-1RA counseling data			
% counseled (if qualify)	220 (35.0%)	104 (41.6%)	324 (36.4%)
If counseled—Who	PCP: 142 (64.5%)	PCP: 67 (64.4%)	PCP: 209 (64.5%)
	Endo: 47 (21.4%)	Endo: 24 (23.1%)	Endo: 71 (21.9%)
	Cardio: 10 (4.55%)	Cardio: 1 (0.96%)	Cardio: 11 (3.40%)
	GI/Bariatrics: 15 (6.82%)	GI/Bariatrics: 4 (3.85%)	GI/Bariatrics: 19 (5.86%)
	Other∗: 6 (2.72%)	Other∗: 8 (7.69%)	Other∗: 14 (4.32%)
If not counseled—No PCP?	73 (17.8%)	28 (19.2%)	102 (18.0%)
If No PCP—Avg derm visits	7.82 (2-35)	7.39 (2-40)	7.71
If No PCP—Other providers (Ps +HS together)			
0-1	24		
2-4	16		
>4	33		

∗ Other includes OBGYN, Psych, Nephrology, EM, Rheum, Derm (*n* = 2).

Psoriasis and HS are chronic inflammatory skin diseases closely associated with metabolic syndromes.^1^ In this cohort, although most patients met eligibility criteria for GLP-1RA therapy, only 36.4% received counseling and 22.1% were prescribed a GLP-1RA, indicating that these agents are underrecognized as treatment options for this population. Nearly one-quarter of eligible patients without a PCP were followed primarily by dermatology, highlighting an opportunity for dermatologists to close this gap by recognizing GLP-1 eligibility, initiating counseling, or facilitating referral to primary care or endocrinology. This study is limited by its retrospective, single-center design and use of EHR data, which may incompletely capture counseling documentation and disease severity. Nonetheless, findings suggest that increasing awareness of GLP-1RAs among dermatologists could expand options for disease management for patients with Psoriasis and HS.^4^

## Conflicts of interest

None disclosed.
